# Cervical spine osteoradionecrosis or bone metastasis after radiotherapy for nasopharyngeal carcinoma? The MRI-based radiomics for characterization

**DOI:** 10.1186/s12880-020-00502-2

**Published:** 2020-09-01

**Authors:** Xi Zhong, Li Li, Huali Jiang, Jinxue Yin, Bingui Lu, Wen Han, Jiansheng Li, Jian Zhang

**Affiliations:** 1 Department of Medical Imaging, Affiliated Cancer Hospital & Institute of Guangzhou Medical University, Guangzhou, 510095 China; 2 Department of Otolaryngology, The Third Affiliated Hospital of Guangzhou Medical University, Guangzhou, 510150 China; 3 Department of Cardiovascularology, Tungwah Hospital of Sun Yat-Sen University, Dong cheng East Road, Dong guan, 523110 Guangdong China; 4 Department of Radiation Oncology, Affiliated Cancer Hospital & Institute of Guangzhou Medical University, Guangzhou, 510095 China

**Keywords:** Magnetic resonance imaging, Nasopharyngeal carcinoma, Radiotherapy, Osteoradionecrosis, Radiomics

## Abstract

**Background:**

To develop and validate an MRI-based radiomics nomogram for differentiation of cervical spine ORN from metastasis after radiotherapy (RT) in nasopharyngeal carcinoma (NPC).

**Methods:**

A radiomics nomogram was developed in a training set that comprised 46 NPC patients after RT with 95 cervical spine lesions (ORN, *n* = 51; metastasis, *n* = 44), and data were gathered from January 2008 to December 2012. 279 radiomics features were extracted from the axial contrast-enhanced T1-weighted image (CE-T1WI). A radiomics signature was created by using the least absolute shrinkage and selection operator (LASSO) algorithm. A nomogram model was developed based on the radiomics scores. The performance of the nomogram was determined in terms of its discrimination, calibration, and clinical utility. An independent validation set contained 25 consecutive patients with 47 lesions (ORN, *n* = 25; metastasis, *n* = 22) from January 2013 to December 2015.

**Results:**

The radiomics signature that comprised eight selected features was significantly associated with the differentiation of cervical spine ORN and metastasis. The nomogram model demonstrated good calibration and discrimination in the training set [AUC, 0.725; 95% confidence interval (CI), 0.622–0.828] and the validation set (AUC, 0.720; 95% CI, 0.573–0.867). The decision curve analysis indicated that the radiomics nomogram was clinically useful.

**Conclusions:**

MRI-based radiomics nomogram shows potential value to differentiate cervical spine ORN from metastasis after RT in NPC.

## Background

Nasopharyngeal carcinoma (NPC) is a unique malignancy with distinct geographic and racial distribution differences. It is particularly prevalent in South-Eastern Asia, Northern Africa, and Southern China [1]. With the application of radiochemotherapy, the local control of NPC has been prominently improved [2]. Osteoradionecrosis (ORN) is a common complication of NPC after radiotherapy (RT), which frequently occurs in the mandible, maxilla, and skull base [3, 4]. Recently, RT induced ORN has been drawn much more attention. However, ORN of the cervical spine only has been described in several case reports and few retrospective studies [5–10].

As the clinical treatment difference between ORN and metastasis, antibiotic administration, sequestrectomy, or hyperbaric oxygen therapy for ORN and RT or chemotherapy for bone metastasis, so it is crucial to distinguish ORN from metastasis [10]. Because biopsy of a cervical spine lesion is risky, and pathologic specimens are seldom available in clinical practice, MRI plays a key role in the diagnosis of cervical spine ORN. However, cervical spine ORN may be misinterpreted as bone metastasis due to its similar clinical and imaging presentation with metastasis [6, 8, 9]. Thus, the accurate diagnosis of cervical spine ORN still remains to be challenging.

Radiomics are involved in the transformation of conventional medical images (MRI, CT, and PET/CT) into analyzable quantitative parameters extracted by data characterization algorithms. Radiomics has been widely applied to differentiate benign and malignant tumors [11, 12], predict tumor grading [13], lymph node metastasis [14, 15], tumor recurrence [16, 17], and patients survival [18, 19]. Besides, MRI-based radiomics has also been used to assess the effects of age on trabecular bone structure and osteoporosis [20], the spatial heterogeneity of the lumbar vertebral bone marrow [21], and subchondral bone alterations of knee osteoarthritis [22]. Especially, MRI-based radiomics features may be used to assess the early structural change of femoral head after RT in prostate cancer [23], and identify vertebral bone marrow metastases in patients with malignancy [12, 24, 25].

Radiomics analysis allows the calculation of quantitative texture parameters to reflect lesions’ histopathological features, which may provide potential value for differentiating benign and malignant diseases. To our knowledge, no comprehensive radiomics-based study has been addressed for the differentiation of ORN from metastasis to date. In this study, we aimed to explore the value of MRI-based radiomics to differentiate of cervical spine ORN from metastasis in NPC.

## Methods

### Patients

**Fig. 1:**
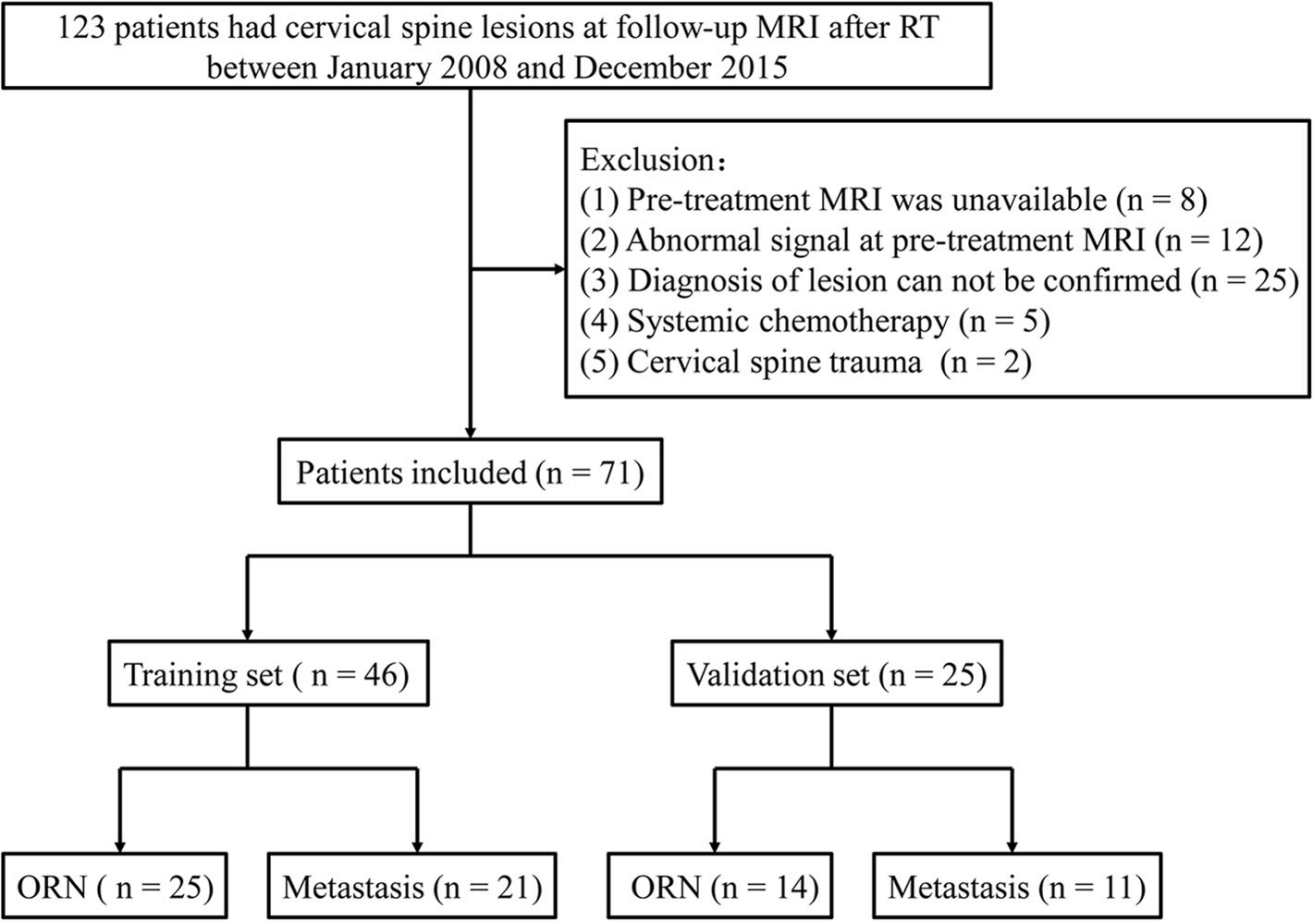
Flowchart of the study population

This retrospective study was approved by the institutional review board at Affiliated Cancer Hospital & Institute of Guangzhou Medical University, and the requirements of patients’ informed consent were waived. Between January 2008 to December 2015, clinical, pathologic, and radiological data of 6451 consecutive NPC patients after RT were reviewed. A total of 123 patients that showed emerging cervical spine lesions on follow-up MRI were selected for further analysis. The inclusion criteria were as follows: (a) underwent pre-treatment MRI and showed no abnormal signal changes in the cervical spine; (b) after lesion detection, histopathology assessment or MRI follow-up at least 6 months were performed for confirming the nature of the lesions; (c) no distant metastasis apart from cervical spine that necessitated systemic chemotherapy, because the chemotherapy may alter imaging features of the cervical spine lesion; (d) no history of cervical spine trauma during follow-up. According to the inclusion criteria, 52 patients were excluded due to the following reasons:(1) Pre-treatment MRI was unavailable (*n* = 8); (2) showed abnormal signal changes in the cervical spine at pre-treatment MRI (*n* = 12); (3) insufficient MRI was performed to confirm the diagnosis of the cervical spine lesions (*n* = 25); (4) receive systemic chemotherapy due to distant metastasis (*n* = 5); (5) underwent cervical spine trauma (*n* = 2). Patients’ inclusion flowchart was displayed in Fig. 1.

Consequently, 71 patients were enrolled in this study, 46 NPC patients (ORN, *n* = 30; metastasis, *n* = 16) gathered from January 2008 to December 2012 were assigned to the training set, and 25 NPC patients (ORN, *n* = 14; metastasis, *n* = 11) gathered from January 2013 to December 2015 were assigned to the validation set.

### MR image acquisition, segmentation and radiomics feature extraction

MR images were acquired using a 1.5-T system unit (Intera Achieva; Philips). The MRI protocol included an axial turbo spin echo (TSE) T1-weighted, an axial TSE T2-weighted, a coronal short time inversion recovery (STIR) sequence, and an axial and a sagittal contrast-enhanced TSE T1-weighted sequence. Contrast-enhanced T1WI was performed after intravenous administration of 0.1 mmol/kg gadopentetate dimeglumine (Magnevist, Bayer Schering). Details of the MRI acquisition were showed in Supplemental Materials (Table S1).

**Fig. 2:**
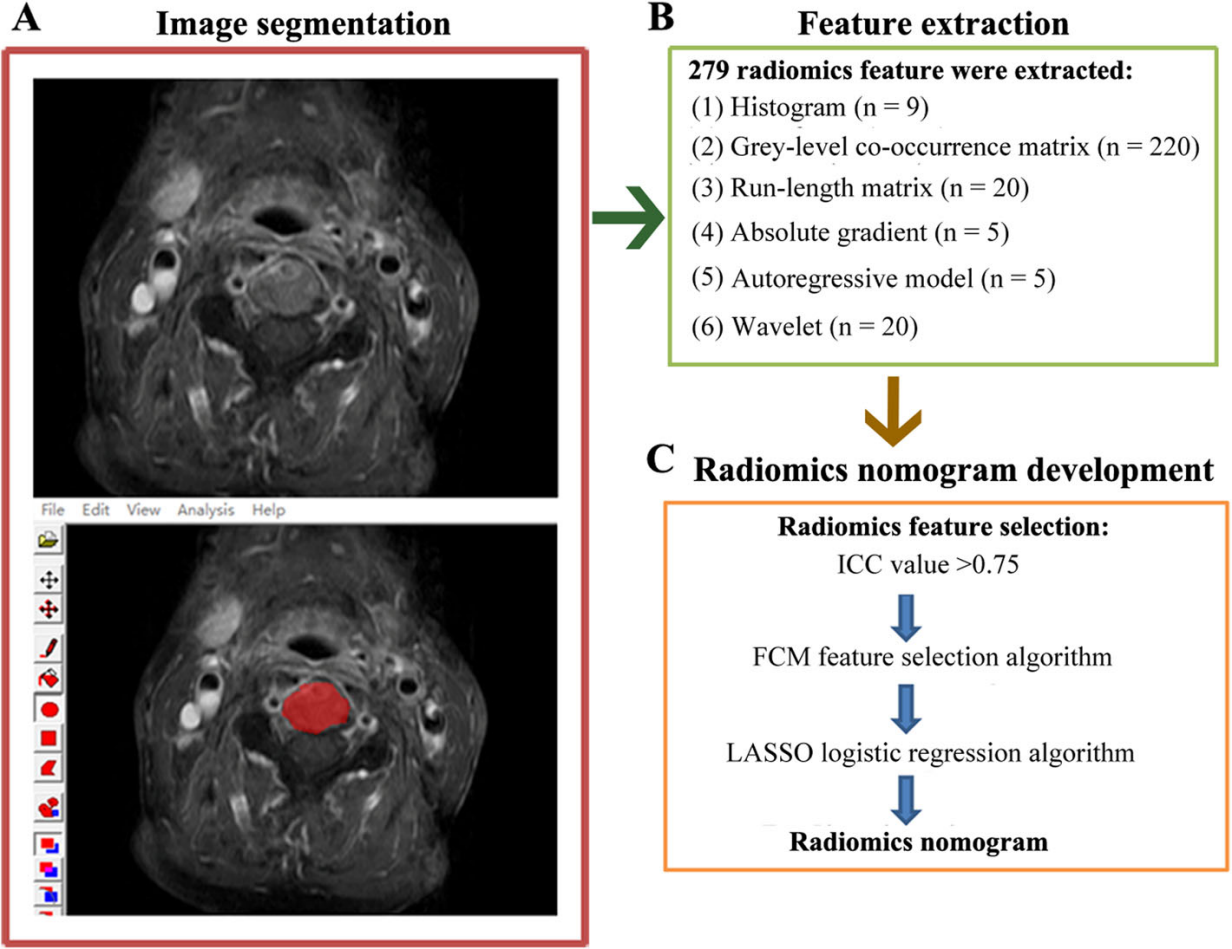
The workflow of the radiomics signature development. **a** the image derived from our hospital, and patients’ informed consent was waived by the institutional review board, a region of interest (ROI) was manually delineated in the largest cross-sectional area of the lesion at CE-T1W image using a software package MaZda 4.6. **b** Feature extraction, a total of 279 radiomics features derived from six statistical image descriptors were extracted. **c** Radiomics nomogram development, features with ICC values greater than 0.75 were selected for subsequent procedure, the remaining features were reduced by using a combination feature selection algorithm of FCM, and then a radiomics nomogram was constructed using the LASSO algorithm

All lesions showed contrast-enhancement, thus axial enhanced T1-weighted images were retrieved from PACS in the “.dicom” format for image feature extraction. Segmentation for regions of interest (ROIs) was performed using a software package MaZda 4.6 (URL: http://www.eletel.p.lodz.pl/programy/mazda/). Before ROIs placement, the gray-level of image was normalized by adjusting image intensities in the range ofu ± 3σ (u, gray-level mean; σ, gray-level standard deviation) [26, 27]. All lesions ROIs were manually delineated in the largest cross-sectional area of lesion (Fig. 2a). In total, 279 radiomics features derived from six statistical image descriptors (Histogram, Grey-level co-occurrence matrix, Run-length matrix, Absolute gradient, Autoregressive model and Wavelet) were extracted (Fig. 2)b. Details of radiomics feature information are in the Supplementary Data (Table S2).

The inter-observer reproducibility of radiomics feature extraction was estimated using interclass correlation coefficients (ICC). The ROI segmentation was performed independently by two radiologists experienced in skeleton MRI interpretation (J.X.Y. and B.G.L. with 10 years of experience). An ICC value > 0.75 indicates good agreement of the feature extraction [14, 28].

### Feature selection and radiomics nomogram development

A radiomics nomogram was constructed in the training set. To identify the most discriminating radiomics feature for differentiating cervical spine ORN from metastasis, feature selection was performed before radiomics signature development. ICC was calculated for the 279 radiomics features, and those features that showed ICC value greater than 0.75 were selected for subsequent procedure. Then the remaining features were reduced by using a combination feature selection algorithm (combination of fisher coefficient [FC], classification error probability combined with average correlation coefficients (CEP+ACC) and mutual information [MI]; FCM) that comprised 30 radiomics features with the most discriminative ability [27, 29].

The least absolute shrinkage and selection operator (LASSO) logistic regression algorithm using10-fold cross-validation based on minimum criteria was adopted for final feature selection for radiomics nomogram development [15, 28]. A formula was created using a linear combination of the selected features that were weighted by their respective LASSO coefficients; then a radiomics nomogram was constructed based on the radiomics score calculated by formula that reflected the possibility of ORN. The procedure of feature selection and radiomics nomogram development was showed in Fig. 2c. The calibration of the nomogram was assessed using a calibration curve, and the Hosmer–Lemeshow test was performed to assess the goodness-of-fit of the nomogram [30]. The diagnostic efficiency of the nomogram for discrimination of ORN and metastasis was assessed by ROC analysis in the training set, and the diagnostic sensitivity and specificity was also calculated.

### Validation of the radiomics nomogram

Validation of the radiomics nomogram was accomplished with the validation set. A radiomics score was calculated for each lesion in the validation set using the formula constructed in the training set. The diagnostic efficiency and calibration of the nomogram model were also assessed in the validation set.

### Clinical utility of the radiomics nomogram

To assess the clinical use of the nomogram, we used the decision curve analysis (DCA) to calculate the net benefits for a range of threshold probabilities in the combined training and validation set. The net benefit is identified as the proportion of true positives minus the proportion of false positives, weighted by the relative harm of false-positive and false-negative results [31].

### Reference standard

The pathological assessment was performed for only one ORN patient. The reference standard without pathological assessment was based on the MRI and clinical follow-up for confirming the diagnosis of the lesions [9, 10]. Lesions with progressive enlargement that presented as soft-tissue masses were identified as bone metastasis. Lesions that shrank or remained stable on MRI for more than 6 months without further treatment were interpreted as ORN. If a lesion’s diagnosis could not be identified based on the follow-up procedure, it would be eliminated.

### Statistical analysis

LASSO logistic regression was performed by using R statistical software (version 3.3.1, http://www.rproject.org/), the “glmnet” package was adopted. Nomogram construction and calibration plots were performed using the “rms” package, and the Hosmer–Lemeshow test was conducted using the “generalhoslem” package. DCA was performed using the “dca.R.” Other statistical analysis was performed using the SPSS 16.0 (SPSS Inc., Chicago, IL, USA), the overall performance was determined by assessing the area under the receiver operating characteristic (ROC) curve (AUC). Mann–Whitney U test and Pearson chi-square test (or Fisher test) were used for continuous and categorical variables, respectively. Statistical tests; *P* < 0.05 indicated statistical significance.

## Results

### Patient characteristics

**Fig. 3:**
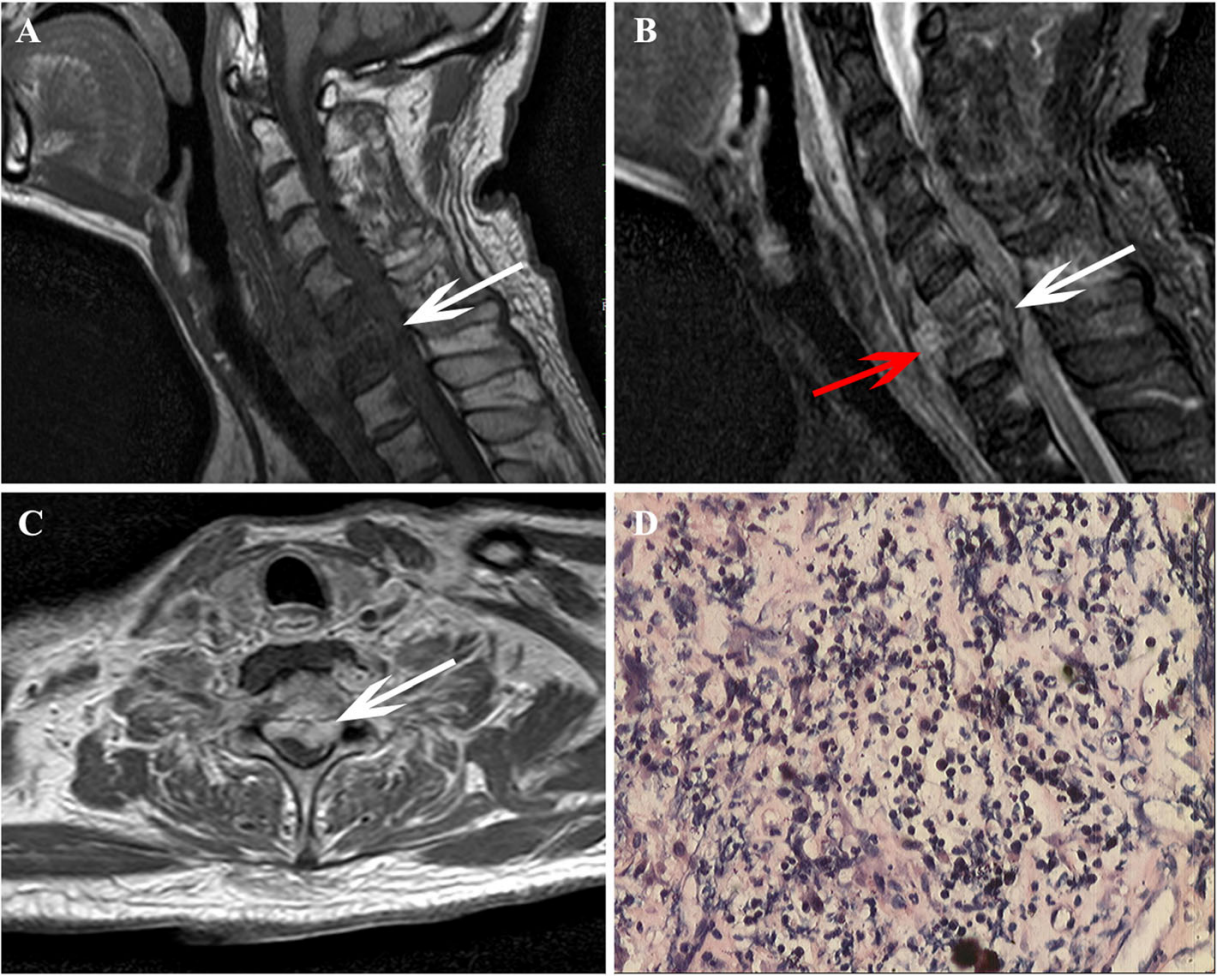
Images in a patient after radiotherapy that was diagnosed with cervical spine ORN by surgery pathology, these images derived from our hospital and patients’ informed consent were waived by the institutional review board. **a** Sagittal T1-weighted image shows hypointensity change in C6, C7 vertebral body, and paravertebral soft tissue, and the spinal canal is pressed (white arrow). **b** Sagittal FS T2-weighted image shows hyperintensity change in C6, C7 vertebral body and paravertebral soft tissue, and the spinal canal (white arrow) and prevertebral endorhachis are pressed (red arrow). **c** Axial CE-T1W image shows marked enhancement of C6 alike soft mass, and the lesion protrudes into the spinal canal (white arrow). **d** Pathological assessment shows inflammatory cell infiltration was identified in the lesion, without malignancy

**Fig. 4:**
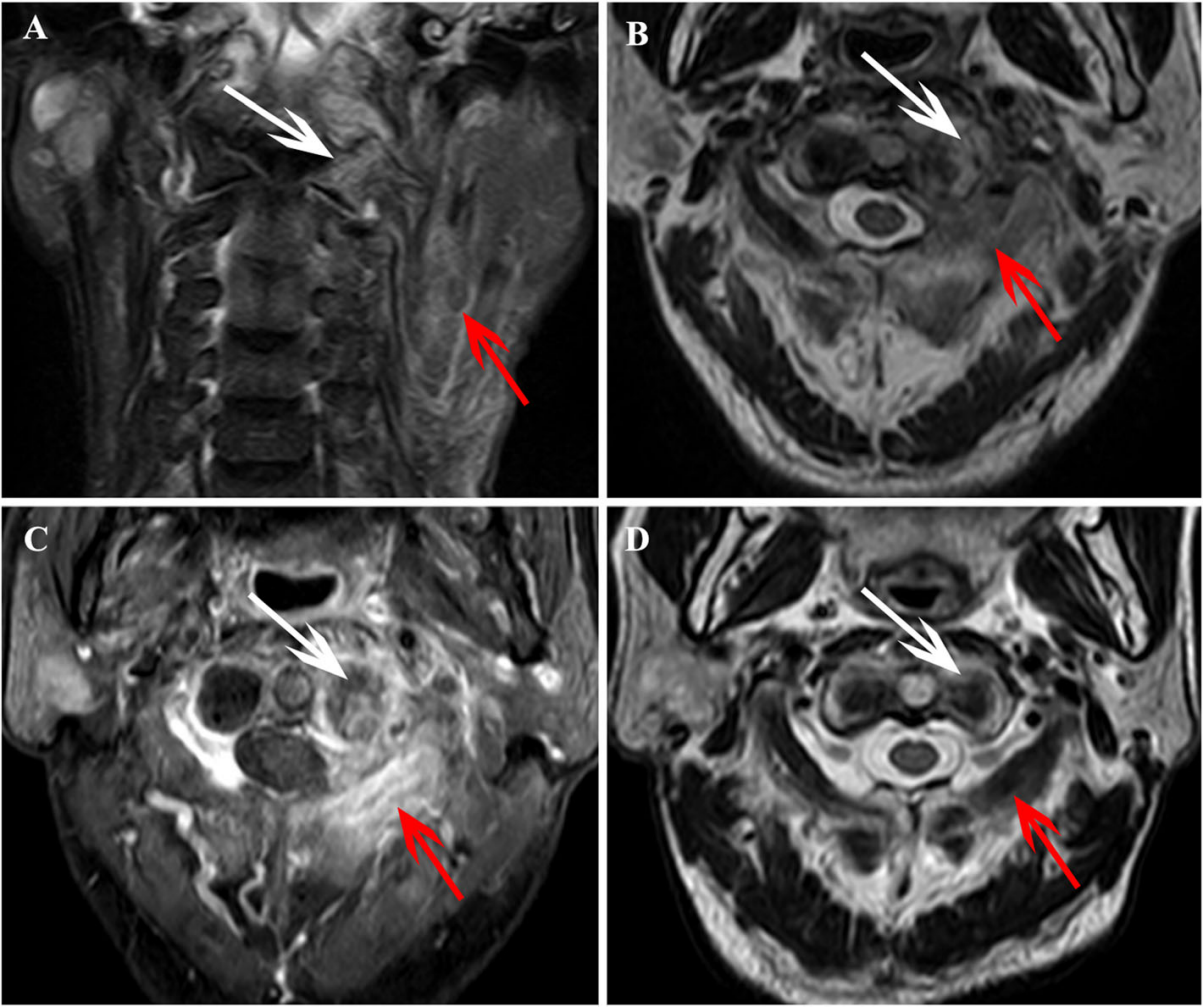
Images in a patient who was diagnosed with ORN of the cervical spine after radiotherapy for NPC, these images derived from our hospital, and patients’ informed consent was waived by the institutional review board. **a** Coronal FS T2-weighted image shows hyperintensity in the left aspect of C1 (white arrow) and shows muscle edema in the left neck (red arrow). **b** Axial T2W image shows hyperintensity in the left aspect of C1 (white arrow) and paravertebral muscle edema (red arrow). **c** Axial enhanced T1W image shows irregular endplate destruction and enhancement in the left aspect of C1 (white arrow), and shows marked enhancement in bilateral paravertebral muscles (red arrow). **d** At MRI follow-up examination after 10 months, the Axial T2W image shows the area of hyperintensity in the left aspect of C1 (white arrow) and paravertebral muscle edema (red arrow) has significantly shrunken

Only one patient underwent cervical spine surgery and diagnosed as ORN (Fig. 3), the diagnosis of the other 70 patients was based on the MRI follow-up process (Fig. 4). The training set showed similar baseline clinical characteristics with that of validation cohorts (*P* > 0.05), except for the frequency of cervical lymphadenopathy (*P* < 0.001). The patients’ detailed characteristics are summarized in Table 1.

**Table 1:** Characteristics of patients in training set and validation set

Characteristics	Training set (*n* = 46)		*P* value	Validation set (*n* = 25)		*P* value
	ORN	Metastasis		ORN	Metastasis	
Number of patients (n)	25 (54.3%)	21 (45.7%)		14 (56.0%)	11 (44.0%)	
Gender						
Male	19 (76.0%)	15 (71.4%)	0.725	11 (78.6%)	8 (72.7%)	0.739
Female	6 (24.4%)	6 (28.6%)		3 (21.4%)	3 (27.3%)	
Age (years)	50.0 ± 10.8	49.1 ± 11.7	0.790	51.8 ± 10.7	48.9 ± 10.5	0.735
Over stage^a^						
I	2 (8.0%)	1 (4.8%)	0.886	1 (7.1%)	1 (9.1%)	0.901
II	10 (40.0%)	7 (33.3%)		4 (28.6%)	2 (18.2%)	
III	8 (32.0%)	6 (28.6%)		6 (42.9%)	4 (36.4%)	
IVA	3 (12.0%)	4 (19.0%)		2 (14.3%)	2 (18.2%)	
IVB	2 (8.0%)	3 (14.3%)		1 (7.1%)	2 (18.2%)	
Involvement multiple lesions	19 (76.0%)	16 (76.2%)		10 (71.4%)	9 (81.8%)	
Overall RT dose (Gy)	69.5 ± 12.3	67.4 ± 17.8	0.617	73.2 ± 9.6	73.0 ± 10.9	0.637
Concurrent chemotherapy	22 (88.0%)	20 (95.2%)	0.373	13 (92.9%)	10 (90.9%)	0.476
Interval time from first RT to lesion detection (mouth)	36.1 ± 26.3	41.3 ± 25.6	0.498	31.3 ± 22.5	32.1 ± 16.2	0.060
Cervical lymphadenopathy^b^	2 (8.0%)	7 (33.3%)	0.033	0 (0.0%)	4 (36.4%)	0.006
Radiation-induced brain necrosis	2 (8.0%)	1 (4.8%)	0.654	1 (7.1%)	0 (0%)	0.275

^a^, According to the 7th UICC/AJCC staging system; ^b^, short axis larger than 1 cm on axial images; *ORN* Osteoradionecrosis;

*RT* Radiotherapy

### Number and locations of ORN and metastasis

The number and locations of lesions are shown in Table 2. Based on the reference standard, a total of 95 cervical spine lesions were identified in training set (ORN, *n* = 51; metastasis, *n* = 44), and 47 lesions were identified in validation set (ORN, *n* = 25; metastasis, *n* = 22). ORN most frequently occurred in the upper cervical spine (C1/C2) (Fig. 4), which accounted for 47.1% (24/51) of all ORNs in the training set and 13/25 (52.0%) of all ORNs in the validation set.

**Table 2:** Number and locations of the cervical spine ORN and metastasis in training set and validation set

Locations	Training set		Validation set	
	ORN	Metastasis	ORN	Metastasis
C1	12	3	5	2
C2	13	5	8	3
C3	6	8	4	4
C4	5	6	2	4
C5	8	10	3	5
C6	5	8	3	3
C7	2	4	0	1
Total	51	44	25	22

*ORN* Osteoradionecrosis;

### Radiomics nomogram model construction

**Fig. 5:**
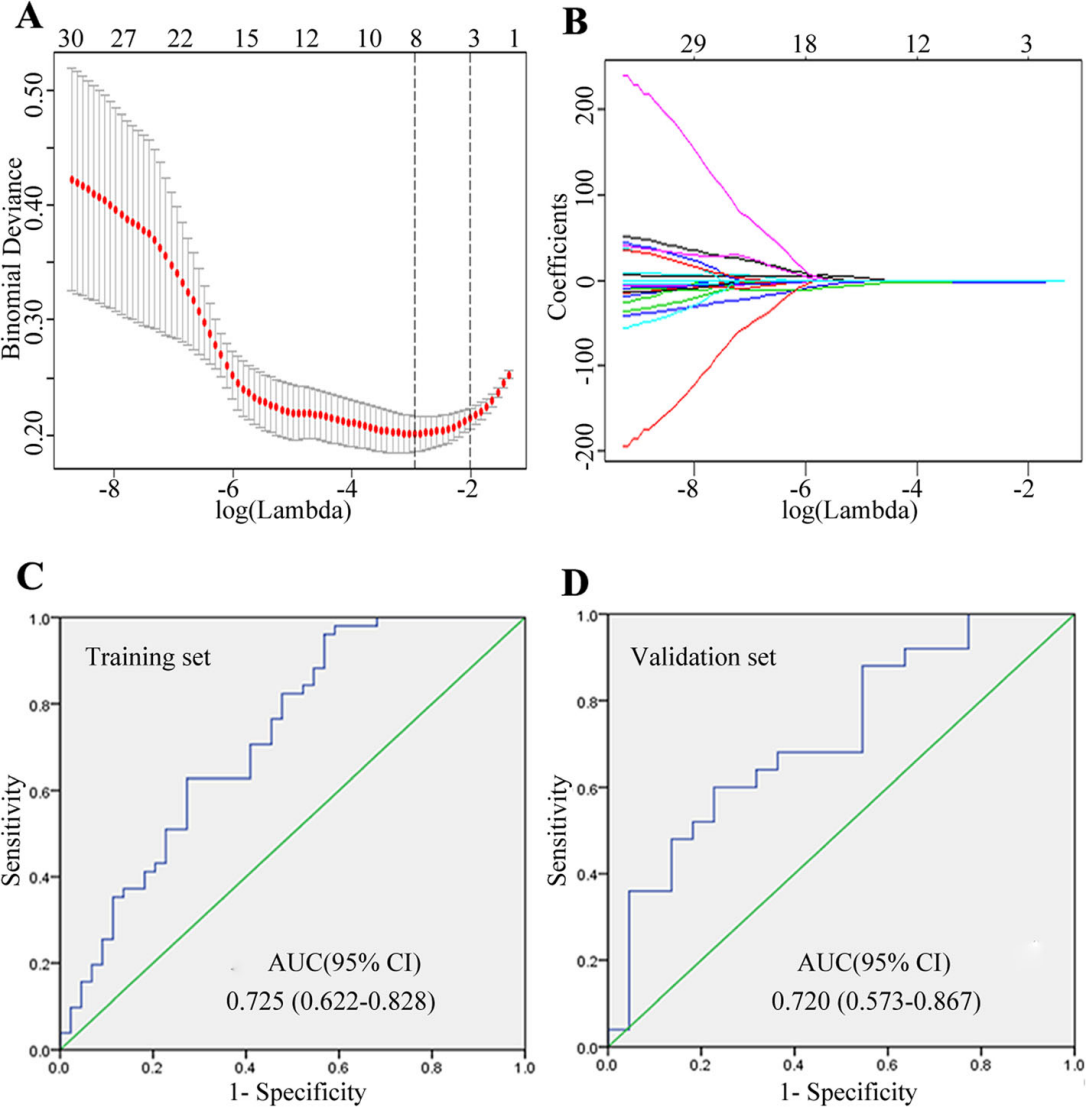
Feature selection using the LASSO logistic regression algorithm and the diagnostic efficiency of the radiomics signature. **a** Selection of the tuning parameter (λ). The LASSO logistic regression model was used with penalty parameter tuning that was conducted by 10-fold cross-validation based on minimum criteria. The y-axis indicates binomial deviances, and the lower x-axis indicates the log (λ). Numbers along the upper x-axis represent the average number of predictors. Red dots indicate average deviance values for each model with a givenλ, and vertical bars through the red dots show the upper and lower values of the deviances. The vertical black lines define the optimal values of λ, where the model provides its best fit to the data. The optimal value of log (λ) = − 2.894 resulting in 8 nonzero coefficients were selected. **b** LASSO coefficient profiles of the 30 texture features, the dotted vertical line was plotted at the value selected using 10-fold cross-validation in **a**. **c ~ d** Diagnostic efficiency of radiomics signature using ROC analysis in the training set **(C)** and validation set **(d)**

A radiomics nomogram was developed in the training set. In total, 279 radiomics features were extracted from CE-T1W images. Of these features, 186 features showed good agreement (ICC > 0.75) were selected for further reduction using the FCM feature selection algorithm. After feature selection, 30 radiomics features were remained for subsequent LASSO algorithm analysis, and these features from the two measurers were averaged for the subsequent analysis. Using LASSO logistic regression analysis, eight features were determined for the construction of the radiomics signature (Fig. 5a and b), and 87.5% (7/8) of the features derived from GLCM, these features and their coefficients were shown in Table 3. Radiomics score was calculated for each lesion by using a formula resulting from the eight features weighted by their coefficients. The formula was expressed as follow:

$$ Y=1.29+\left(0.0000324\times \mathrm{Vertl}\_\mathrm{RLNonUni}\right)-\left[0.289\times \mathrm{S}\;\left(5,\hbox{-} 5\right)\mathrm{DifVarnc}\right]-\left[0.00964\times \mathrm{S}\;\left(5,5\right)\mathrm{SumOfSqs}\right]-\left[0.00186\times \mathrm{S}\;\left(5,0\right)\;\mathrm{SumOfSqs}\right]-\left[0.267\times \mathrm{S}\left(4,4\right)\mathrm{SumOfSqs}\right]-\left[0.00215\times \mathrm{S}\left(3,-3\right)\mathrm{SumOfSqs}\right]-\left[0.00301\times \mathrm{S}\left(0,2\right)\mathrm{SumOfSqs}\right]+\left[0.0414\times \mathrm{S}\left(1,0\right)\mathrm{SumVarnc}\right]. $$

**Table 3:** Calculation formula for radiomics signature

Parameters	Textural groups	Coefficients
Intercept		1.29
Vertl_RLNonUni	Run-length matrix	0.0000324
S(5,-5)DifVarnc	GLCM	−0.289
S(5,5)SumOfSqs	GLCM	−0.00964
S(5,0)SumOfSqs	GLCM	−0.00186
S(4,4)SumOfSqs	GLCM	−0.267
S(3,-3)SumOfSqs	GLCM	−0.00215
S(0,2)SumOfSqs	GLCM	−0.00301
S(1,0)SumVarnc	GLCM	0.0414

### Performance and validation of the radiomics nomogram

**Fig. 6:**
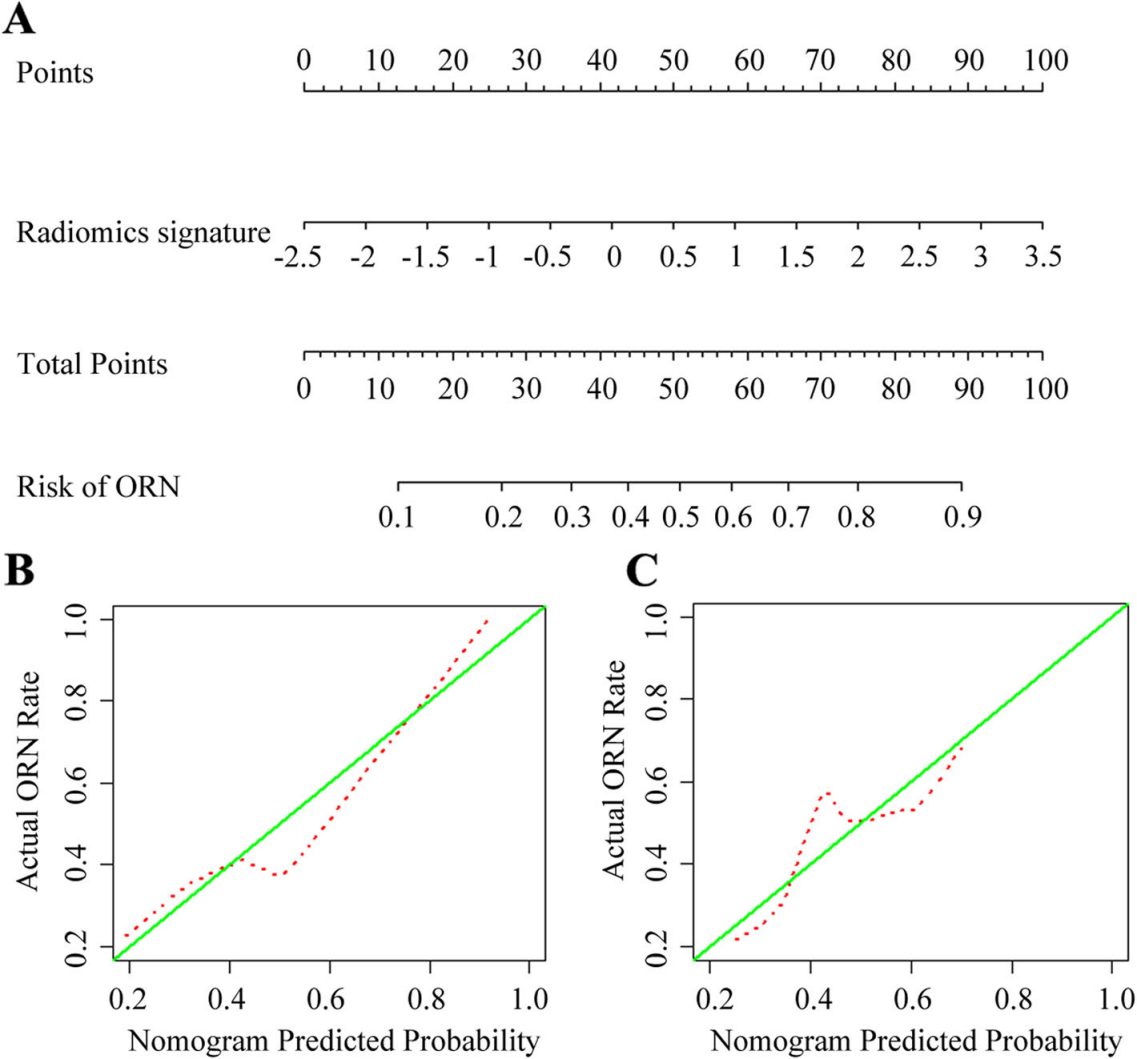
Radiomics nomogram development and calibration. **a** Radiomics nomogram was developed in the training set based on the radiomics. **b** Calibration curves of the radiomics nomogram in the training set. **c** Calibration curves of the radiomics nomogram in the training set. Calibration curves depict the calibration of the nomogram in terms of an agreement between the predicted risk of ORN and observed outcomes. The 45^0^ green lines represent a perfect prediction, and the dotted red lines represent the predictive performance of the nomogram. The closer the dotted red line fit is to the green line, the better predictive accuracy of the nomogram

Based on the radiomics scores in the training set, a nomogram was constructed (Fig. 6a), and the calibration curve of the nomogram for both training set and validation set were plotted (Fig. 6b and c). Using ROC analysis in the training set, the nomogram model showed good discriminatory ability in the differentiation of cervical spine ORN and metastasis, with the AUC of 0.725 (95% [CI], 0.622–0.828) (Fig. 5c), the sensitivity of 84.3% (43/51), the specificity of 61.4% (27/44). Application of the model in the validation set still showed good diagnostic efficiency, with the AUC of 0.720 (95% [CI], 0.573–0.867) (Fig. 5d), the sensitivity of 80.0% (20/25), the specificity of 64.0% (14/22). The diagnostic performance of the radiomics nomogram in the training and validation sets as shown in Table 4.

**Table 4:** Diagnostic performance of radiomics mode in the training and validation sets

Variables	Az (95%CI)	Sensitivity	Specificity	Accuracy
Training set	0.725 (0.622,0.828)	84.3% (43/51),	61.4% (27/44)	73.7% (70/95)
Validation set	0.720 (0.573,0.867)	80.0% (20/25)	64.0% (14/22)	72.3% (34/47)

### Clinical use of radiomics nomogram

**Fig. 7:**
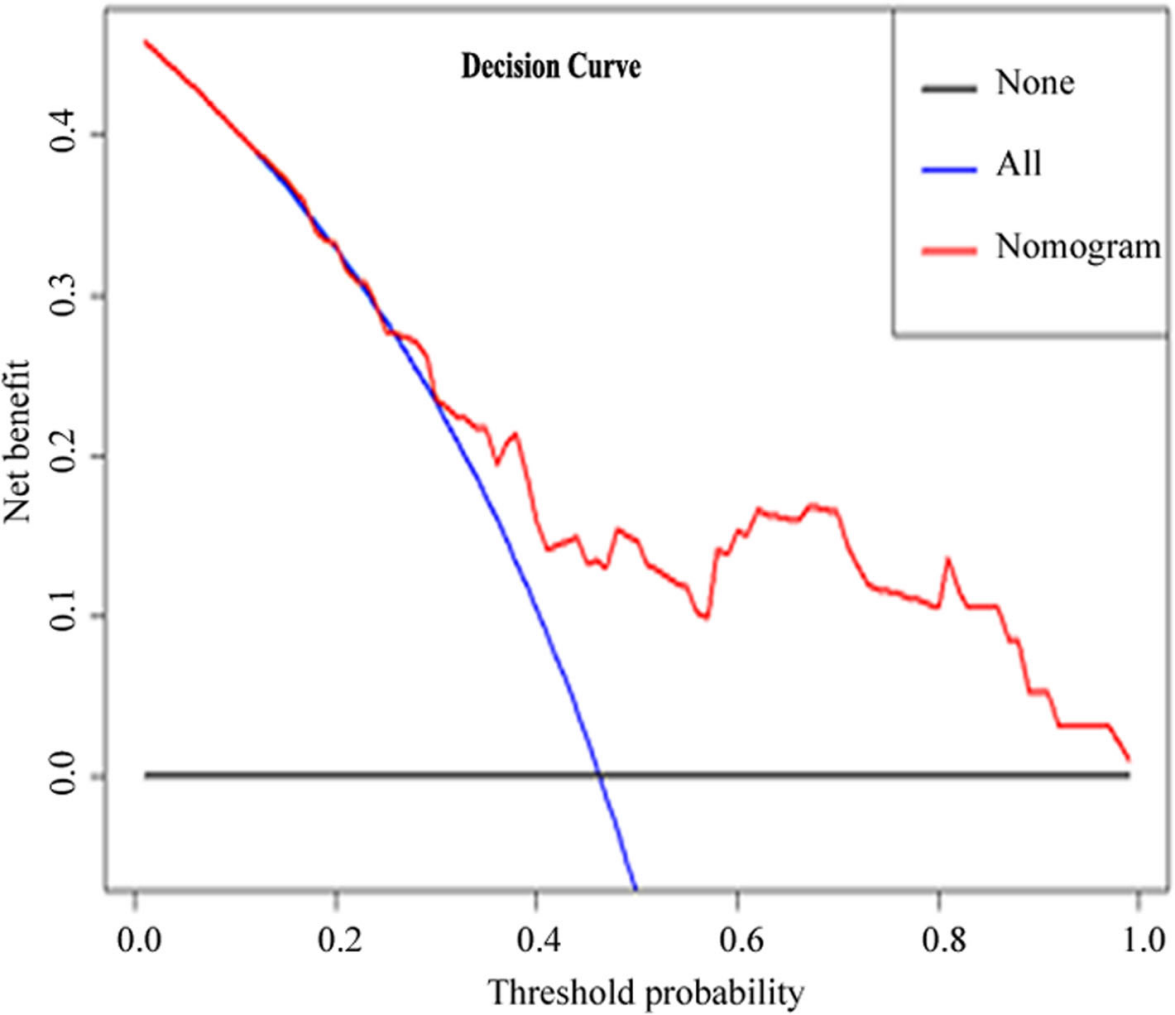
Decision curve analysis (DCA) for the radiomics nomogram. The y-axis represents the net benefit. The red line represents the radiomics nomogram. The blue line represents the hypothesis that all lesions were ORN. The black line represents the hypothesis that no lesion was ORN. The net benefit was calculated by subtracting the proportion of all patients who are false positive from the proportion who are truly positive, weighting by the relative harm of forgoing treatment compared with the negative consequences of unnecessary treatment. DCA indicated that if the threshold probability of a lesion for diagnosis as ORN is > 12%, in this case, using the radiomics nomogram to diagnose ORN adds net benefit than either the treat-all-patients scheme or the treat-none scheme. For example, if the threshold probability is 50%, then the net benefit is 0.147 when using the radiomics nomogram to make a decision

The decision curve analysis (DCA) for the radiomics nomogram is presented in Fig. 7. The DCA showed that if the threshold probability of a lesion for diagnosis as ORN is > 12%, using the radiomics nomogram to diagnose ORN adds net benefit than either the treat-all-patients scheme or the treat-none scheme.

## Discussion

In this study, we have developed and validated an MRI-Based radiomics nomogram for the differentiation of cervical spine ORN from metastasis in patients with NPC after RT. We found that the radiomics nomogram showed good calibration and discrimination, with an AUC of 0.725 in the training set and 0.720 in the validation set, respectively. Our results indicated that MRI-Based radiomics may be used as a noninvasive tool for differentiating cervical spine ORN from metastasis after RT.

NPC is one of the highly invasive and metastatic head and neck cancer, and cervical spine ORN is a serious complication in NPC after RT [4, 5]. Accurately diagnose cervical spine ORN and distinguish it from bone metastasis is quite important, because an improper diagnosis may create excessive and hurtful chemoradiotherapy for patients. Recently, MRI has been recommended as a very useful technique for the identification of benign and malignant vertebral diseases [32, 33]. There are several studies have clarified the value of MRI for diagnosis of ORN, displayed that cervical spine ORN could be misdiagnosed as bone metastasis, because cervical ORN could show soft-tissue masses and present abnormal enhancement [6, 9, 10].

Recently, radiomics features have shown great prospect in the identification of malignant and benign bone marrow diseases, including differentiation of primary malignant and benign bone tumors [12], discrimination of benign and malignant vertebral compression fractures [25], and differentiation metastatic and completely responded sclerotic bone lesion [24]. Particularly, a recent study demonstrated that MRI-based radiomics features could be used to assess the early structural change of femoral head after RT and may show potential value to predict RT-induced femoral fractures [23]. However, the value of radiomics features in the characterization of ORN and metastasis is still unclear. In this study, we found eight radiomics features based on CE-T1WI were significantly associated with the differentiation of cervical spine ORN and metastasis. Meanwhile, in line with previous studies performed in other fields [12, 26, 28], we found these discriminative features that selected to differentiate ORN and metastasis were most frequently derived from GLCM categories.

In the present study, we firstly explored the performance of an MRI-based radiomics nomogram for differentiation of cervical spine ORN from metastasis. We found that the radiomics nomogram constructed in the training set showed good discrimination efficiency, with an AUC value of 0.725, the sensitivity of 84.3% and the specificity of 61.4%. Then, we verified the value of this radiomics nomogram in the validation set and still showed good discrimination, with an AUC of 0.720, sensitivity of 80.0%, specificity of 64.0%. Thus, MRI-based radiomics may be a non-invasive imaging biomarker for differentiating cervical spine ORN from metastasis after RT. The results supported previous studies in which MRI-based radiomics could be applied to differentiate vertebral metastasis from benign lesions [25, 34].

The major issue for the clinical application of the nomogram is based on the need to interpret individual net benefits. Nevertheless, the discrimination efficiency and calibration may not acquire the clinical consequences of a particular level of discrimination or degree of miscalibration [35, 36]. To address this issue, we assessed the clinical use of the nomogram by using decision curve analysis (DCA) in the combined training and validation set. This new strategy offers insight into clinical outcomes based on the threshold probability, from which the net benefit could be obtained [14, 28]. In this study, DCA indicated that if the threshold probability of a lesion for diagnosis as ORN is > 12%, in this case, using the radiomics nomogram to diagnose ORN adds net benefit than either the treat-all-patients scheme or the treat-none scheme. In line with a previous study showed that the threshold probability was > 10% for prediction of lymph node metastasis in colorectal cancer [15].

Our study had some limitations. First, this was a retrospective study performed in a single center with a relatively small sample size. Thus, multicenter validation is needed to achieve strong evidence for its clinical application. Second, as described in previous studies [6, 9, 10], pathologic confirmation for cervical spine ORN and bone metastasis was not available attributed to the relatively high risks related to biopsy of the cervical spine (eg, injury to the vertebral artery or the cervical spinal cord). Third, only radiomics features are selected to construct a nomogram model, because the object of this study was cervical spine lesion, considering the fact that patients could involve single or multiple lesions, include patients’ clinical factors could create selection bias.

## Conclusions

MRI-based radiomics nomogram may serve as a noninvasive visual diagnostic tool for differentiation cervical spine ORN from metastasis in patients with NPC after RT. Multicenter external validation is necessary to acquire high-level evidence for its clinical application.

## Supplementary information

**Additional file 1: Table S1.** MRI sequences and parameters. **Table S2.** Radiomics features calculated by using MaZda4.6**.**

## Data Availability

The datasets used and/or analyzed during the current study available from the corresponding author on reasonable request.

## Abbreviation

RT: Radiotherapy
NPC: Nasopharyngeal carcinoma
ORN: Osteoradionecrosis
LASSO: Least absolute shrinkage and selection operator
ROC: Receiver operating characteristic curve
AUC: Area under the receiver operating characteristic curve
GLCM: Grey-level co-occurrence matrix
ROI: Regions of interest
FC: Fisher coefficient
CEP+ACC: Classification error probability combined with average correlation coefficients
MI: Mutual information
FCM: Combination of FC, CEP+ACC and MI
CE-T1WI: Contrast-enhanced T1-weighted image
