# Supplementary material for: Cervical spine osteoradionecrosis or bone metastasis after radiotherapy for nasopharyngeal carcinoma? The MRI-based radiomics for characterization
Source: BMC Med Imaging. 2020 Sep 1;20:104. doi: 10.1186/s12880-020-00502-2 (PMC7466527; doi:10.1186/s12880-020-00502-2)
Supplement: Supplementary file 1 — Additional file 1: Table S1. MRI sequences and parameters. Table S2. Radiomics features calculated by using MaZda4.6. [file 12880_2020_502_MOESM1_ESM.docx]

**Details of MRI acquisition**

MR images were acquired using a 1.5-T system unit (Intera Achieva; Philips Healthcare, Best, The Netherlands) with a 16-channel head-neck combined coil. The MRI protocol included an axial turbo spin echo (TSE) T1-weighted sequence, an axial TSE T2-weighted sequence, a coronal short time inversion recovery (STIR) sequence, and an axial and a sagittal contrast-enhanced TSE T1-weighted sequence. Contrast-enhanced T1WI was performed after intravenous administration of 0.1 mmol/kg gadopentetate dimeglumine (Magnevist, Bayer Schering). Details of the MRI acquisition were showed in **Table S1**

**Table S1:** MRI sequences and parameters

| **Sequence** | **FS** | **TR/TE (ms)** | **Gap (**mm**)** | **ST(mm)** | **FOV(cm)** | **Matrix** |
| --- | --- | --- | --- | --- | --- | --- |
| Axial TSE T1-weighted | No | 545/14 | 4 | 4 | 23 | 328 × 220 |
| Axial TSE T2-weighted | No | 3193/80 | 5 | 5 | 23 | 228 × 185 |
| Coronal STIR | Yes | 3224/165 | 5 | 5 | 26 | 312 × 163 |
| Axial contrast-enhanced TSE T1-weighted | Yes | 545/14 | 4 | 4 | 23 | 328 × 220 |
| Sagittal contrast-enhanced TSE T1-weighted | Yes | 545/14 | 4 | 4 | 26 | 328 × 220 |

*FS*, fat suppression; *TR*, repetition time; *TE*, echo time; *STIR,* short time inversion recovery *; ST*, slice thickness;

*FOV*, field of view

**Radiomics feature extraction**

279 radiomics features derived from six statistical image descriptors (Histogram, Co-occurrence matrix, Run-length matrix, Absolute gradient, Autoregressive model and Wavelet) were extracted. The texture features details are described in **Table S2**.

**Table S2: Radiomics features calculated by using MaZda4.6.**

| Statistical descriptors | Features |
| --- | --- |
| Histogram | A total 9 of features :Mean, skewness, kurtosis, variance, percentiles 1%, 10%, 50%, 90%, and 99% |
| Co-occurrence matrix | A total of 220 features :Angular second moment, contrast, correlation, sum of squares, inverse difference moment, sum average, sum variance, sum entropy, entropy, difference variance, and difference entropy. Features are computed for 5 between-pixel distances (1, 2, 3, 4, 5) and for 4 different directions (vertical, horizontal, 0, 135). |
| Run-length matrix | A total of 20 features: Run-length nonuniformity, gray-level nonuniformity, long-run emphasis, short run emphasis, and fraction of image in runs. Features are computed for 4 different directions (vertical, horizontal, 0, 135). |
| Absolute gradient | A total of 5 features : Mean, variance, skewness, kurtosis, and percentage of pixels with nonzero gradient |
| Autoregressive model | A total of 5 features : Theta 1 (θ1) Theta 2 (θ2), Theta 3 (θ3), Theta 4 (θ4), and Sigma (σ) |
| Wavelet | A total of 20 features: WavEn (wavelet energy). Features are computed at 5 scales within 4 frequency bands LL, LH, HL, and HH. |
